# Oral health experience of individuals with eating disorders

**DOI:** 10.1186/s40337-024-01117-6

**Published:** 2024-10-09

**Authors:** Ulrica Gidlund, Tove Hasselblad, Pernilla Larsson-Gran, Yvonne von Hausswolff-Juhlin, Göran Dahllöf

**Affiliations:** 1 Department of Prosthetic Dentistry, Public Dental Service, Folktandvården Stockholms Län AB, Stockholm, Sweden; 2 Department of Dental Medicine, Karolinska Institutet, Stockholm, Sweden; 3 Department of Dental Science, Malmö University, Malmö, Sweden; 4 Center of Oral Rehabilitation, Folktandvården Östergötland, Norrköping and Linköping, Sweden; 5 Center for Psychiatry Research, Department of Clinical Neuroscience, Karolinska Institutet, Stockholm, Sweden; 6 Stockholm Health Care Services, Region Stockholm, Sweden; 7 Center for Oral Health Services and Research, Mid-Norway, TkMidt, Trondheim, Norway

**Keywords:** Anorexia nervosa, Bulimia nervosa, Dental erosion, Eating disorders, Thematic analysis

## Abstract

**Background:**

Evidence on how persons with and in remission from an eating disorder experience their oral health is limited. Dental treatment in Sweden today is often postponed until medical rehabilitation has been completed, but this carries risks. For the patient, the risk is severely impaired oral health and additional suffering, and for both society and the patient, higher costs than might have been necessary.

**Methods:**

Ten female informants aged 21–51 years (mean age = 36.7, standard deviation 12.7) in remission from an eating disorder with a median duration of 12.5 (range 4–25) years of illness, were questioned in semi-structured interviews about their perceptions of oral health. All participants had been referred to a specialist dental clinic and needed oral rehabilitation. 10% of the patients had been diagnosed with anorexia nervosa and 90% with bulimia nervosa. All had been in remission from the eating disorder for at least one year. Transcripts of the interviews were analyzed with thematic analysis using an inductive approach.

**Results:**

One overarching theme emerged from the analysis: dental damage persisted as a visible, lingering scar during remission of the eating disorder, reminiscent of the disease and its consequences. The three major themes identified were (1) Physical impact, (2) Psychological impact, and (3) Impact on daily living. The first major theme included erosive tooth wear and impaired oral function and aesthetics. Interviewees described the second as feelings of stigma, guilt, shame, anxiety, and worry, in particular concerning self-inflicted dental damage through self-induced vomiting. The last major theme covered avoidance strategies such as limiting smiling and laughing and minimizing social situations such as eating with others, pursuing a wanted career, and meeting a partner.

**Conclusions:**

The participants in this study expressed a profound negative impact on daily life and a two-fold burden of stigma of having suffered from both an eating disorder and poor oral health.

## Abstract (plain language summary)

Research has established a link between eating disorders and poor oral health, such as erosive tooth wear. One knowledge gap, however, is how patients with and after recovery from an eating disorder experience their oral health. In Sweden, dental treatment is often postponed until after medical rehabilitation, but this carries risks. For the patient, the risk is severely impaired oral health and additional suffering, and for both society and the patient, higher costs than might have been necessary. The self-reports of the patients in their interviews witnessed to their suffering from two sources: the diagnosis of a mental disorder and poor oral health. Many reported feelings of stigma, shame, and guilt over, for example, self-induced vomiting, which was a predominant factor in dental damage. Tooth damage emerged as a visible, lingering scar that reminded the patient of the disease long after remission.

## Background

**Fig. 1:**
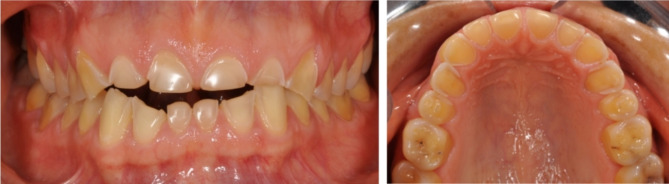
Permanent dentition showing severe dental erosion in a 40-year-old woman, suffering from an eating disorder for 20 years, including bulimia nervosa with self-induced vomiting. (**A**) Frontal view (**B**) Occlusal view of upper jaw

Eating disorders (EDs) considerably impair physical health and psychosocial functioning and are both deadly and costly mental disorders [1–3]. Wanting to control weight, body shape, and eating play a key role in the origin and maintenance of EDs [1]. In Western settings overall, 5.5–17.9% of women and 0.6–2.4% of men have experienced an ED by early adulthood [4]. Six main feeding and eating disorders: anorexia nervosa, bulimia nervosa, binge eating disorder, avoidant restrictive food intake disorder, pica, and rumination disorder are now recognized in The Diagnostic and Statistical Manual of Mental Disorders, Fifth Edition (DSM-5) [5]. Many symptoms of these disorders imply a high risk for oral health, causing caries, loss of teeth, and dental erosion [6, 7] (Fig. 1.). The symptoms of a long-term ED (e.g., self-starvation, self-induced vomiting, rumination, and chewing on food without swallowing) often vary over time, and cumulatively, they comprise a high risk of poor oral health, causing great suffering and high costs for both the patient and society when untreated. The Dynesen et al. study on the experiences and perceptions of oral health and dental care among patients with EDs revealed gaps in our knowledge of the impact of EDs on oral health and of adequate treatment protocols in dental care for this patient group [8]. Furthermore, patients in the Patterson-Norrie et al. study with experience of having an ED expressed barriers such as embarrassment, shame, and high costs in contact with dental care. They also expressed need for a greater emphasis on oral health prevention while still ill [9]. Thus, there is need to improve the dental treatment of patients with EDs throughout the course of their illness. Based on this, the aim of this study was to broaden our understanding of how patients, with eating disorders and after recovery, experience their oral health. To achieve this, the research question was set as: “How do individuals with and after recovery from an eating disorder experience their oral health?”

## Methods

### Pilot study

Before developing the interview guide, the study group carried out a pilot study. Two patients who had been referred to the Department of Prosthetic Dentistry at Eastman Institute in Stockholm, Sweden, and had experiences of an ED were recruited. One needed dental treatment, and one had recent experience of extensive oral rehabilitation. These participants, not included in the present study, were chosen to cover the spectrum of experiences from before, during, and after an oral rehabilitation. The interview questions concerned patient perceptions of their oral health in relation to their ED, expectations before extensive oral rehabilitation, and the experience of oral rehabilitation itself.

### Interview guide

The interview guide comprised three open-ended items: (1) What can you tell me about the health of your teeth? (2) What is your experience of dental care and dental treatment? (3) What are your hopes, expectations, and fears, now before oral rehabilitation?

### Participants

Participants were recruited from patients referred for dental treatment at Eastman Institute between 31 January 2022 and 18 January 2023. Eastman Institute is a specialist dental clinic that operates under the auspices of the Swedish Public Dental Service in Stockholm. This qualitative study is part of a larger research project studying oral rehabilitation in patients with experience of EDs. Purposive sampling was used to select the research persons for this study. Inclusion criteria were that participants were 18 years or older, had experience of an ED that had been diagnosed by a psychiatrist according to the DSM-5 [10], were in remission from the ED, needed oral rehabilitation, and had good verbal skills in Swedish for describing their experiences. The treating dentist (UG) invited the patients to participate in the study after having performed a dental examination at the dental clinic. The interviews took place after the patients had agreed to the dental treatment plan but before the start of or in the initial stage of oral rehabilitation.

### Exclusion of eligible participants

Recruitment of participants to this interview study was difficult, even among participants who, without hesitation, had agreed to participate in the larger (clinical intervention) studies of the project. Six participants who fulfilled the inclusion criteria were so reluctant to meet a psychologist to talk about their ED that they were excluded. Another three eligible participants were considered too vulnerable to participate (by UG) and were not invited to the study to eliminate the risk of triggering a relapse of their ED.

### Data collection

Ten female participants were admitted to the study, aged 21–51 years (mean age = 36.7, standard deviation 12.7). The participants had a median duration of 12.5 (range 4–25) years’ experience of an ED, were in remission, and in need of oral rehabilitation, with a median of 19.5 (range 6–26) teeth in need of restoration. The diagnoses of the patients included dental caries, dental erosion with enamel loss, orofacial pain, impaired esthetics, and lowered occlusal height. Hence, all needed extensive oral rehabilitation and had been referred to a specialist dental clinic by their regular dentist. A psychiatrist (YHJ) diagnosed 10% of the participants with previous anorexia nervosa and 90% with previous bulimia nervosa. All participants were medically rehabilitated and had been in remission for at least 1 year, in agreement with published duration criteria [11]. Five participants were employed in the work force: a physician (1), a stockbroker (1), a teacher (1), a self-employed, in fashion (1), and in the health food industry (1). Three informants were students: dance (1), law (1), and florist (1). One participant had interrupted her studies in architecture due to the ED and was receiving sickness benefits. The interviews took place in a conference room at Eastman Institute, separate from the dental clinic. A psychologist (TH) with previous experience of conducting interviews in dental care studies and with no part in the patient’s medical or dental treatment conducted the interviews. TH let the informant take the lead and only asked follow-up questions when necessary or when the discussion died down. Based on the structure of the interview setting with open ended questions the interviews varied in time depending on the participants’ ability to express themselves regarding their experiences. Thus, the interviews lasted between 13 and 81 min and were recorded on tape. A language service company (Spoken, www.spokencompany.se) transcribed the interviews in Swedish. GD (who has previous experience of qualitative research) and UG then proofed the transcripts by comparing them to the audio files [12, 13]. All participants appear under pseudonyms in the interviews, the transcribed text, and this publication.

### Data analysis

We analyzed the transcripts from the interviews using thematic analysis according to Braun and Clarke [14]. The analysis followed an inductive approach: deriving meaning and creating themes from the data without preconceptions. The research team consisted of a psychiatrist, a psychologist, and three dentists: two senior consultants of prosthodontics and one pediatric dentist and searched for themes and patterns across the complete set of data. Following Braun and Clarke, the first step in analysis was to become familiarized with the data by reading and re-reading the transcribed interviews and reviewing the recordings several times. In the initial coding phase, the content was summarized and categorized into codes that expressed key concepts in the data. Next, the various codes were grouped into themes. To identify a theme, a satisfactory answer to the question “What is this expression an example of?” had to be evident and appear as a repeated pattern of interest in the data. Thematic maps where then constructed to visualize the relationship between themes before applying the themes back to the total set of data for further interpretations. Since many of the quotes cover several major themes, one quote can be used to illustrate, for instance, both physical and psychological impact.

## Results

An overarching theme that emerged from the data was a visible, lingering scar. Many informants expressed how, after remission from the ED, the poor oral health continued to have an impact on their lives. This overarching theme consisted of three major themes: (1) Physical impact, (2) Psychological impact and (3) Impact on daily living as shown in Table 1.

**Table 1:** Consequences of having experience of an eating disorder and poor oral health

Overarching theme	Major themes	Subthemes
A visible, lingering scar	Physical impact	Orofacial pain Tooth breakdown Shortened teeth Impaired aesthetics Focus on teeth
	Psychological impact	Stigma Shame Guilt Worry Anxiety Low self-esteem Disaster thinking
	Impact on daily living	Avoidance of normal behaviors: Smiling Laughing Socializing Eating with others Difficulty meeting a partner Limited career choices

### The overarching theme

The overarching theme emerged, among others, from informant remarks such as the below.

*I think about it (the eating disorder) all the time when I look at them (the teeth)*,* and teeth are something you see all the time*,* so it becomes more noticeable than if it were something else that you can hide more… You’ve fought so hard to get well and when you’re well… it (the teeth) becomes a reminder*,* and it would be very nice to avoid that reminder and be able to restore…to what you looked like before.*

#3, 22 years

*I’m 50 soon and it feels so unnecessary*,* so sad*,* that something that happened when I was young lives on in such a way that you can’t really get free from it. Besides (the teeth)*,* I’m perfectly healthy today*,* but this is what’s left and then it’s almost 30 years later.*

#8, 50 years

#### Major theme 1: physical impact

All participants described extensive experience with the physical impacts on oral health, such as severe breakdowns in tooth substance and dental restorations. They also reported consequences of these physical impacts, such as pain.

… *I noticed that when I had eating disorders*,* they (the teeth) got weaker and weaker… I have very fragile teeth*,* with fractures of both teeth and restorations. As soon as I have bitten into a peach or eaten something I can get piece of a tooth in my mouth. It’s very difficult to know that teeth can break anytime.*

#2, 49 years

*They (the teeth) have been shortened*,* so the enamel is very thin*,* I have a hard time eating an apple.*

#3, 22 years

Participants reported severe pain connected with food intake and even during tooth brushing.

*After the eating disorder*,* I noticed that it started to hurt a lot more when I ate ice-cream or if I drank hot tea.*

#4, 21 years

*Some pain is very easy to repress*,* while other pain is very difficult to repress*,* and toothache*,* it has become like a matter of habit to me…*

#2, 49 years

The informants also expressed impaired esthetics due to shortened and fractured teeth, transparency of the teeth and yellow tooth color.

*I don’t think my smile is so nice anymore because I have short teeth*.

#4, 21 years

*As for my teeth*,* I am very unhappy. I feel like I’ve ruined them*,* I absolutely don’t like the way they look*,* the color*,* basically everything about the teeth*.

#6, 32 years

*I think they (the teeth) are very yellow and colored*,* ugly color*,* and everyone else is bleaching their teeth*,* but I have been told that I cannot do that… because they are too fragile.*

#2, 49 years

All these impairments, stated by the interviewed, are expressions of severe erosive tooth wear, causing loss of the enamel, exposure of the dentin and changes in the anatomy and color of the teeth due to lack of enamel. The physical impact on oral health led to a constant awareness of the teeth, a greater focus on oral health, and a fear of continuing deterioration of the teeth. The participants also reported difficulties eating and ingesting sufficient high-quality food due to pain, sensitivity, tooth breakdown, and food impaction, when masticating.

#### Major theme 2: psychological impact

Participants reported a two-fold stigma of having suffered from a mental disorder and living with poor oral health. Several participants described the aftereffects of an eating disorder like battling a war with oneself.

*It’s like I’m two. First*,* we have this high achiever*, and then it’s like Dorian Gray’s portrait*. You know,* Oscar Wilde. There is Dorian and there is the portrait… There is the real me and there is me projected outward. The feeling inside of me has been like a wormy rotting corp*.

#1, 49 years

*Oscar Wilde, The picture of Dorian Gray, 1891

*I haven’t had any teeth lately; I’ve almost felt like a bag lady.*

#2, 49 years

All participants expressed guilt over having caused the dental damage themselves and felt that the visible tooth damage persisted as a constant reminder of the ED and its consequences.

*When I am over the eating disorder*,* the damage to my teeth is still there. And when I look at my teeth…I ask: what I’ve done to myself?*

#4, 21 years

*It is the eating disorders*,* the bulimia and the vomiting that is the fundamental factor in it. There’s a lot of shame in me because I know it’s me who has caused it in a way*,* even if it was not me (the eating disorder)*,* it is still me.*

#6, 32 years

*That you have destroyed something that you have naturally*,* which has been very good and very functional… knowing that you have knowingly or unknowingly destroyed them is even more a burden.*

#5, 46 years

Shame was another central feeling that all participants reported: Shame about having had an ED. Shame about having suffered from bulimia nervosa instead of anorexia nervosa since bulimia nervosa includes self-cleaning (purging), which three participants described as a reprehensible and disgusting behavior. Two participants defined having bulimia nervosa as being a “failed anorectic”, which added to the shame. The guilt about not being able to take control over the ED led to self-hatred. The two-fold burden of fear had a severe negative impact on quality of life: fear of others finding out about the mental disease and fear of other noticing one’s poor oral health.

*I was afraid of being exposed*,* of someone saying: “But God*,* what is this (the tooth damage)?” I felt a deep shame and embarrassment over everything I had done… and because I keep an outward facade and I am well-functioning*,* highly educated*,* raising a family*,* married. On paper*,* everything looks very good. Family dinners*,* good girl*,* but inside the feeling was dreadful.*

#1, 49 years

*…there is a lot of shame*,* there is an extreme amount of guilt when I think about my teeth… I also feel like hating myself at the same time because*,* I’m not happy at all…*.

#6, 32 years

Low self-esteem, linked to the poor oral health, was another feeling the participants expressed.

…*it is easy to feel bad about yourself when you’re ashamed of your teeth because they’re so obviously visible and you need to use them too.*

#2, 49 years

The experience of these participants, of living with the daily burden of stigma, guilt, shame, and self-hatred, was negatively reinforced by repeated unmet needs in contact with dental care.

…*I have asked if I can get help (at the dentist) “but no*,* there is no possibility for you” … you must accept that your teeth look like shit.*

#2, 49 years

…*I feel self-hatred… because I know that I am a fundamental factor in it. Because if I had never started vomiting or had never had an eating disorder*,* then my teeth would not look the way they do today.*

#6, 32 years

Living with constant anxiety and worry about the risk of tooth deterioration which would require future extensive and expensive dental treatment also affected the participants negatively, causing obsessive thoughts and disaster thinking. Many participants also expressed hopelessness when dental treatment was not provided.

*…Okay*,* it won’t get any better. It just goes down*,* down*,* and down*,* I can’t seem to get help. I understood myself that at some point I will probably have to make (dental) crowns*,* and that affected me as well. I panicked as that it is not something I will be able to afford…*

#7, 23 years

*It’s a great deal of anxiety because I have the dental damage I have*,* and yes*,* it affects me a lot. You learn to live with it and get used to the situation you have with your teeth*,* but as soon as something happens… a small infection or something completely normal and safe for everyone else… I always expect the worst*,* that there will be huge problems.*

#10, 51 years

#### Major theme 3: impact on daily living

All participants in this study reported different strategies for managing daily life while suffering from poor oral health. Limiting smiling and laughing by different means was one strategy used to avoid showing their teeth.

*I haven’t dared to smile in so many years……I have always smiled with my mouth shut…I’ve learned to talk by not moving my lips so much…Sometimes I may even have covered my mouth with my hand.*

#1, 49 years

*I don’t want to smile. Always trying to smile with my mouth closed*,* always*,* covering my mouth. I don’t want to laugh either*,* even though I am a very happy person… I don´t want to be in pictures because… there is a chance that my teeth will be visible*,* and I don’t want that… There is so much that prevents me from living a normal everyday life.*

#6, 32 years

Avoiding socialization and eating with others were other reported strategies. Such measures inhibited participants from showing happiness about being in remission from their mental disorder since they could not freely act out their emotions.

*I have a very strong memory from eight grade. We were really going to smile*,* and I just didn’t dare. I kind of smiled the best I could with my lips*,* but not with my teeth. There were also several people who started asking: How am I doing? Am I depressed?… I kind of started recovering from my eating disorder and was on the road to recovery. But the teeth caused me not to …*

#7, 23 years

None of the participants in this study expressed dental fear; on the contrary, many participants had sought dental care several times, sometimes over a period of 10–15 years. Each time, they expressed extensive dental treatment need, but they were not offered treatment. All study participants were strongly motivated to maintain a good oral health.

…*I remember the first time I threw up*,* the first time I understood that now this had turned into binge eating; into a bulimia… I read and googled*,* and it said that the teeth can be destroyed*,* so I called my dentist and made an appointment*,* because I just needed to know if I had any injuries. The dentist did absolutely nothing. The only thing she did was prescribe mouthwash for me and said: Rinse your mouth after every time you vomit.*

#4, 21 years

Due to their oral health status, which made smiling impossible, two participants expressed limitations in career development and another two, not being able to meet a partner. In summary, these limitations had a considerably negative impact on the psychosocial health and functioning of the participants.

## Discussion

The most important finding in this study is that, even after years of remission, poor oral health persists as a constant reminder of the ED. The tooth damage functioned as a visible, lingering scar that persisted after remission of the ED; this was the overarching theme identified in the data. The three major themes were (1) the physical impact on oral health, (2) the psychological impact of poor oral health, and (3) the impact on daily living.

The physical impacts identified in this study were dental erosion causing pain and tooth sensitivity, brittle teeth prone to breakdown, enamel loss causing reductions in the occlusal vertical dimensions, and impaired aesthetics. These findings are consistent with other studies which reported dental erosion as the most common oral health problem in patients with EDs [7, 15]. The Johansson et al. study found that patients with EDs were 8.5 times more likely to have dental erosion compared to healthy controls [6]. In our study, pain with highly sensitive teeth was more commonly expressed by the younger participants, comparable to other studies on adolescent patients with EDs [16, 17]. The formation of secondary dentin over time may be a reason why the older patients in this study reported fewer symptoms of sensitivity from their teeth. However, the physical impact of impaired aesthetics with shortened, damaged, yellow, and unpleasant looking teeth impaired the patient’s ability to socialize and eat with others, causing a negative impact on oral health-related quality of life, which the Lo Russo study also described [15].

None of the participants in this study expressed dental fear, in contrast to other studies [6, 15]. On the contrary, all participants in this study stated that they had sought dental care several times, sometimes over a period of 10–15 years, and all had an objective need for dental treatment. The participants felt they were overly preoccupied with the condition of their teeth and had a constant fear of dental breakdown, comparable to other patient groups with risk of dental breakdown [18].

All participants in this study reported feeling stigmatized, from having suffered from a mental disorder, which Crisp et al. also described [19], and from living with poor oral health. All participants also expressed guilt due to having inflicted the dental damage on themselves. Some participants described the aftereffect of having an ED like battling a war with oneself and constantly loosing, which Williams & Reid also described [20].

Fear of shame for being exposed as having had an ED was another feeling that was expressed. The shame might have been higher for our study participants than for other groups since the participants were well educated and expectations on them for maintaining good oral health were high.

There is support in the literature for how poor oral health and untreated oral diseases can significantly impact quality of life, including loss of self-esteem and decreased economic productivity [21]. Quality of life can be intangible and difficult to define. “Oral health, although generally not life threatening, can affect the way one eats, speaks, and socializes. Hence, poor oral health can have a profound negative effect on interpersonal relationships, self-esteem, and quality of life” [21, 22].

Several participants also expressed a feeling of being a failed anorectic, which caused even more shame since anorexia nervosa has a higher status in the public in general, and in the community of EDs. The scoping review of Brelet et al. also describes this experience, highlighting the underrepresentation of research on bulimia nervosa and binge-eating disorder compared to the interest in anorexia nervosa [23] The review noted as well that stigma content differs between different EDs [23].

Low self-esteem linked to poor oral health was another feeling the participants expressed. This is consistent with Huff et al. who state that oral health problems affect self-esteem to the same extent as other health related conditions, such as cancer, diabetes, high blood pressure, and asthma [22]. The research group also stated that “The face is the very core of human identity” and that individuals with poor oral health may feel self-conscious and embarrassed, which limits social interaction and communication [22]. Self-image and self-esteem are integral components of one’s ability to reach full psychosocial potential [24], which agrees with our findings.

Poor oral health can interfere with building self-esteem and other types of psychological development; in fact, self-esteem may have more potential for predicting health behavior than other more specific personality measures [24]. However, the effects of deteriorated oral health on self-esteem have not been a priority among health care providers for vulnerable populations, such as patients with EDs. In their study on the experience of Norwegian dentists who treated patients with EDs, Johansson et al. found a lack of knowledge and difficulty meeting the needs of this patient group [25].

The Norwegian dentists expressed difficulties in making contact with, treating, and even getting along with patients with EDs [25]. Other studies confirmed these findings of a lack of knowledge among dental health care professionals regarding meeting the dental needs of this patient group [8, 9]. These results are consistent with the results of the present study where nine of the ten patients interviewed expressed repeated unmet needs in contacts with dental care. How these participants have experienced dental care, and which needs and expectations of dental treatment they have expressed will be described in another study.

## Conclusion

This study has shown how experienced poor oral health caused by an eating disorder affects the lives of participants years after they have been rehabilitated from their medical condition. An eating disorder is a reversible disease, in contrast to the irreversibility of the dental damage. The damaged teeth remain as a visible lingering scar, impossible to hide or neglect, acting as a constant reminder of the disease In this study, the experienced dental damage led to inhibition in social contexts, which negatively affected the quality of life of the participants. The participants also expressed concern about future deterioration of their oral health and worry about high future costs for dental treatment. This study provides important knowledge about the extent of experienced oral health problems in a vulnerable population and how profound the presence of stigma, guilt, and low self-esteem issues are related to oral health.

### Rigor and trustworthiness

All interviews were conducted before, or in the beginning of, the patient’s oral rehabilitation. Thus, with two exceptions, the participants had only met the attending dentist (UG) at one dental visit. However, the fact that the attending dentist who recruited the study participants (UG) was both a clinician and a researcher in this study was addressed during data analysis. A psychologist (TH) with knowledge of dental care and previous experience of conducting interviews, and who had no other part in the patient’s medical or dental treatment, conducted the interviews. A good pre-understanding from the research group representing different medical disciplines (psychiatry, psychology, and dentistry) and with extensive experience of the patient group was considered both an advantage and a disadvantage in the inductive analysis process. The varying backgrounds of the researchers enabled detection of relevant codes and themes in the material, but this might also have caused a disregard of other significant expressions due to differing pre-understandings of both the unit of analysis (individuals with experience of an ED and poor oral health) and the patient group. Throughout the analysis process, the researchers did not seek consensus among the multiple coders. On the contrary, the researchers agreed to disagree, and consistently went back to the transcriptions to find the answer to the true expression of the research question in the data. The decision to participate in the study may have elicited a social desirability bias in study participants. A tendency to cast oneself, consciously or not, in a positive light, or present oneself in agreement with social expectations, may have motivated participants to report fewer or more stigmatizing behaviors and attitudes, and over- or underestimate the significance of poor oral health caused by an ED on quality of life.

### Ethics

The choice of data collection with individual interviews instead of a focus group was made to not expose the disease and dental issues of the study participant. The risk of triggering a relapse of the ED was considered when including participants in the study; thus, some individuals were excluded, although they were included in the oral rehabilitation study. In the event of any concern among the participants within the framework of the study, support was offered at Stockholm’s Center for Eating Disorders. Any adverse events were registered and addressed continuously and reported to the study sponsor (Public Dental Service, Stockholm, Sweden) within 24 h, for immediate intervention.

## Data Availability

No datasets were generated or analysed during the current study.
